# Towards comprehensive and transparent reporting: context-specific additions to the ICF taxonomy for medical evaluations of work capacity involving claimants with chronic widespread pain and low back pain

**DOI:** 10.1186/1472-6963-14-361

**Published:** 2014-08-29

**Authors:** Urban Schwegler, Jessica Anner, Andrea Glässel, Mirjam Brach, Wout De Boer, Alarcos Cieza, Bruno Trezzini

**Affiliations:** Swiss Paraplegic Research (SPF), Nottwil, Switzerland; Department of Health Sciences and Health Policy, University of Lucerne and SPF, Nottwil, Switzerland; asim, Academy of Swiss Insurance Medicine, University Hospital Basel, Basel, Switzerland; Faculty of Social and Human Sciences, School of Psychology, University of Southampton, Southampton, S017 1BJ UK

**Keywords:** International Classification of Functioning, Disability and Health (ICF), Medical evaluation of work capacity, Disability evaluation, Chronic widespread pain, Low back pain, Context-specific additions

## Abstract

**Background:**

Medical evaluations of work capacity provide key information for decisions on a claimant’s eligibility for disability benefits. In recent years, the evaluations have been increasingly criticized for low transparency and poor standardization. The International Classification of Functioning, Disability and Health (ICF) provides a comprehensive spectrum of categories for reporting functioning and its determinants in terms of impairments and contextual factors and could facilitate transparent and standardized documentation of medical evaluations of work capacity. However, the comprehensiveness of the ICF taxonomy in this particular context has not been empirically examined. In this study, we wanted to identify potential context-specific additions to the ICF for its application in medical evaluations of work capacity involving chronic widespread pain (CWP) and low back pain (LBP).

**Methods:**

A retrospective content analysis of Swiss medical reports was conducted by using the ICF for data coding. Concepts not appropriately classifiable with ICF categories were labeled as specification categories (i.e. context-specific additions) and were assigned to predefined specification areas (i.e. precision, coverage, personal factors, and broad concepts). Relevant specification categories for medical evaluations of work capacity involving CWP and LBP were determined by calculating their relative frequency across reports and setting a relevance threshold.

**Results:**

Forty-three specification categories for CWP and fifty-two for LBP reports passed the threshold. In both groups of reports, precision was the most frequent specification area, followed by personal factors.

**Conclusions:**

The ICF taxonomy represents a universally applicable standard for reporting health and functioning information. However, when applying the ICF for comprehensive and transparent reporting in medical evaluations of work capacity involving CWP and LBP context-specific additions are needed. This is particularly true for the documentation of specific pain-related issues, work activities and personal factors. To ensure the practicability of the multidisciplinary evaluation process, the large number of ICF categories and context-specific additions necessary for comprehensive documentation could be specifically allocated to the disciplines in charge of their assessment.

## Background

Medical evaluations of work capacity (MEWC) determine a claimant’s diagnoses and work capacity as the key information for decisions on eligibility for benefits provided by national disability insurances. To ensure a fair eligibility decision process, MEWC should be documented as transparently and comprehensibly as possible [1]. Moreover, MEWC should also be comparable in terms of interrater reliability between the medical experts who are in charge of the assessments [2, 3].

In reality, however, MEWC are often reported in a poorly standardized way [2] and charged with low interrater reliability [4]. Furthermore, in many European countries the determination of a health condition is required as a key criterion for disability benefits eligibility [5], Annex] although health conditions taken by themselves are usually only loosely correlated with work ability limitations [6]. In contrast, modern medical thinking defines disability not simply as the consequence of a health condition but as the result of various biopsychosocial interactions [7]. Hence, transparent MEWC require comprehensive reporting of functional limitations and their determinants, not only in terms of impairments or health conditions but also in terms of contextual factors.

**Figure 1:**
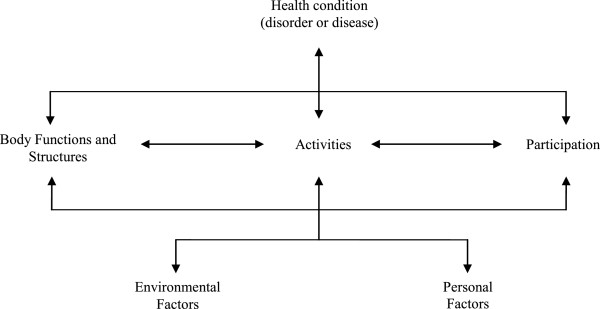
**The comprehensive biopsychosocial framework of the ICF.** Note: Drawn from [7].

The *International Classification of Functioning, Disability and Health* (ICF) [7] provides a comprehensive biopsychosocial framework that conceptualizes functioning as the interplay between a *health condition*, *body functions* and *body structures*, *activities* and *participation* as well as contextual factors, i.e. *environmental* and *personal factors* (see Figure 1). The ICF framework thus appears promising for ensuring transparent reporting in MEWC. The ICF taxonomy is considered the worldwide standard for reporting on functioning and disability and offers a comprehensive spectrum of categories for documenting all components of the framework except personal factors which are not classified. In addressing 362 categories on the second level (e.g. *b280 Sensation of pain*) and up to 1,424 categories on the more specific third or fourth levels (e.g. *b2801 Pain in body part* and *b28013 Pain in back*), the ICF taxonomy provides a common language for standardizing MEWC and enhancing their interrater reliability [8].

Applying the ICF could be particularly beneficial in MEWC of claimants with chronic pain which are in Switzerland often conducted in multidisciplinary settings. The impact of pain on functioning, and thus on work ability, depends on complex biopsychosocial interactions [9]. Therefore, a comprehensive and accurate documentation of functional limitations and their determinants is needed for transparent MEWC involving chronic pain.

So far, no *empirical* studies have examined the comprehensiveness of the ICF taxonomy in covering the core content of MEWC. *Conceptual* papers on the applicability of the ICF in MEWC argue that the ICF is neither useful for describing (in)consistencies and causal relationships between impairments, contextual influences, activity limitations and work ability restrictions, nor for addressing the dynamic development of disability over time [10, 11]. However, since these issues reflect procedural and decisional challenges in MEWC rather than specific aspects related to functioning, they fall beyond the scope of the ICF taxonomy and will not be further dwelled upon in this study.

Yet, the comprehensiveness of the ICF taxonomy in addressing core aspects of *specific contexts* of application has been empirically studied in fields other than MEWC, including work and pain assessments. A need for *context-specific additions* to the ICF was identified with regard to the following four areas.

*(1) Precision* refers to the number of distinct levels of specification within an ICF category [12]. Some categories may not be specific enough for contexts requiring highly accurate reporting of health-related aspects [13]. Existing ICF categories do not allow for describing the pain location or features of pain quality such as pressure, stabbing or rest pain at a sufficient level of detail [14, 15]. Additionally, specific work activities such as “overhead working” or “forward bending stand” cannot be adequately reported with ICF categories [16]. Therefore, when applying the ICF taxonomy for accurate reporting in a specific context, category specifications may be developed for exclusive use in this particular context.

To exploit the maximum precision of the ICF categories for documentation in specific contexts, scholars have advocated using the more accurate third or fourth category levels. For pain assessments, this was mainly motivated by the need to differentiate between specific pain locations [15]. In the work context, the third and fourth category levels allow for distinguishing specific work activities such as sitting or standing [17].

*(2) Coverage* reflects the ability of the ICF taxonomy to comprehensively capture the spectrum of functioning aspects and environmental factors and has formerly been described with the terms *exhaustiveness* or *width* [12]. Some work-related aspects such as “overloading” or “overstressing” can, for instance, not be addressed with ICF categories [18]. To address this issue, items that allow for reporting important context-specific aspects not covered by the ICF could be exclusively generated for a particular context.

*(3) Personal factors* are currently not classified by the ICF taxonomy, although several studies pointed out the need for standardized personal factor categories [19–21]. Psychological aspects that are relevant in the field of chronic pain such as coping strategies, fear-avoidance beliefs or catastrophizing cannot be reported with ICF categories [22, 23]. In the contexts of work and MEWC, it would be important to have categories for the documentation of personal factors such as a claimant’s occupational background, work motivation or expectations regarding return to work [24, 25].

*(4) Broad concepts* such as quality of life or general health were also mentioned as not being classified by the ICF [26]. However, as the ICF taxonomy aims at categorizing specific aspects related to functioning and health rather than overall concepts, this issue appears to be less pertinent.

Applying the ICF taxonomy to code the content of medical reports is one possible way to empirically test its comprehensiveness in the context of MEWC, and to establish aspects that are not addressable with ICF categories at all or not in a sufficiently specific manner. This is, however, based on the assumption that current medical reports do indeed include the crucial aspects of MEWC. In a recent study [27], we found that the ICF Core Sets for chronic widespread pain (CWP) [23], low back pain (LBP) [28] and two major co-morbidities, i.e. depression [29] and obesity [30], cover the *relevant aspects of functioning and environmental factors* in reports on Swiss disability claimants with CWP and LBP to a fair extent. However, the study only focused on second level ICF categories. Moreover, a number of concepts in the reports were not appropriately classifiable with the ICF. A more in-depth analysis of these concepts is necessary to properly establish aspects to be added when applying the ICF taxonomy for comprehensive reporting in MEWC.

The present study aims at providing additions to the ICF exclusively for its application in MEWC involving chronic pain and *not* for an update of the ICF taxonomy in general. Therefore, a *context-specific addition* (or a *specification category*) refers to a complement to the ICF taxonomy for this particular field of application. The four abovementioned *specification areas* (i.e. precision, coverage, personal factors, broad concepts) will serve as a structuring device for presenting the study results.

### Objective

The objective of this study was to identify potential context-specific additions to the ICF taxonomy for its application in MEWC involving CWP and LBP.

### Specific aims

The specific aims were (1) to identify and specify content of medical reports on claimants with CWP and/or LBP not appropriately classifiable with the ICF; and (2) to determine specification categories as well as third and fourth level ICF categories that appear relevant across these reports.

## Methods

### Study design

We conducted a retrospective qualitative and quantitative content analysis of 72 medical reports [31]. In the qualitative part the reports were coded using the ICF, while in the quantitative part a frequency analysis of the coded categories was carried out.

### Ethics

The study was approved by the Ethics Commission of Basel, Switzerland, project number 134/08, and performed in accordance to the Declaration of Helsinki.

### Sample

The available sample included all 209 reports in German that were submitted to the Swiss national disability insurance scheme between February 1^st^ and April 30^th^ 2008, and contained a diagnosis of CWP and/or LBP. We used a selection of *International Classification of Diseases* (ICD-10) codes as inclusion criteria (see Table 1). The reports were selected and anonymized by insurance employees and could include one, two or more medical disciplines.

**Table 1:** **ICD-10 diagnoses included in the sample**

ICD-10 diagnoses for CWP	ICD-10 diagnoses for LBP
F45.0 Somatization disorder	M42 Spinal osteochondrosis (.15-.17, .95-.97)
F45.1 Undifferentiated somatoform disorder	M45 Ankylosing spondylitis
F45.4 Persistent somatoform disorder	M46 Other inflammatory spondylopathies (.0, .1, .2, .3)
F54 Psychological and behavioral factors associated with disorders or diseases classified elsewhere	M47 Spondylosis and (osteo-)arthrosis of spine (.05-.07, .15-.17, .25-.27)
F62.8 Chronic pain personality syndrome	M48 Other spondylopathies (.05-.07, .15-.17, .25-.27)
F32 Mild, moderate and severe depressive episode, with somatic symptoms	M51 Other intervertebral disc disorders (.0, .1)
F33 Recurrent depressive disorder, with somatic symptoms	M53 Other dorsopathies, not elsewhere classified (.25-.27, .3, .86-.87, .96-.97)
F34.1 Dysthymia (in relation with pain)	M54 Dorsalgias (.05-.07, .15-.17, .3, .4, .5, .85-.87)
F43.2 Adjustment disorders	M99 Biomechanical lesions, not elsewhere classified (.03, .13, .23, .33, .43, .53, .63, .73, .83, .93)
M79.7 Fibromyalgia	
R52.2 Other chronic pain	
R52.9 Pain, unspecified	

Note: Drawn from [27].

From this available sample we drew a subsample whose final size was determined based on two criteria: (1) *saturation*, i.e. the assumption that the collected information is sufficient when no new second level ICF category appears in five successive reports analyzed [32, 33]; and (2) *heterogeneity*, i.e. the proportional inclusion of both the relevant medical disciplines of pain assessment (e.g. rheumatology, psychiatry or neurology) and the index conditions (i.e. CWP, LBP) involved in the reports. The heterogeneity criterion was applied to capture the diversity of the concepts relevant in the present context. Taking into account the two abovementioned heterogeneity dimensions a minimum subsample size of 72 reports, representing about one third of the available sample of 209 reports, was determined. The reports were randomly drawn from the available sample and the order in which they were analyzed was randomly determined.

### Analysis

We subdivided the sample into reports with CWP and with LBP diagnoses. Reports including both diagnoses were analyzed twice, once with the pure CWP and once with the pure LBP reports.

### Content analysis

In Switzerland, MEWC are usually documented in free text by medical experts. Reports consist of three main sections that comprehensively address the claimant’s situation. (1) The *socio-medical history* describes the claimant’s occupational, biographical and medical background and his or her functioning in everyday life, including subjective claims regarding impairments and functional limitations. (2) The *medical examination* aims at an objective assessment of functional capacity and documents the expert’s findings regarding the claimant’s physical or mental impairments leading to the final diagnoses. (3) The *work capacity evaluation* provides a synthesis of the two previous sections and an appraisal of the claimant’s work capacity based on his or her functional capacity and diagnoses. In addition, the consistency between subjective claims and objective findings is discussed. Finally, a long-term prognosis is provided and measures to improve the claimant’s work capacity are suggested.

We coded the content of the reports using the ICF and established linking rules [34, 35]. Pre-existing medical records on the claimant were not analyzed. First, we divided the reports into *units of meaning* referring to passages with a common theme (e.g. “the claimant suffers from pain while sitting”). Then, we determined the different *concepts* underlying a unit of meaning (e.g. pain, sitting) and coded them to the most precise ICF category (e.g. *b280 Sensation of pain*, *d4153 Maintaining a sitting position*). To ensure data quality, all reports were coded by two health professionals familiar with the ICF and trained in the linking method. In case of discrepancies, the coders discussed and agreed on their final coding. Any disagreement was resolved by consulting a third subject-matter specialist. Interrater reliability between the two coders was determined based on *percentage agreement* [36].

The coders also assessed whether concepts reflected *limitations* or *barriers* for the claimant (e.g. “the cold weather worsens the claimant’s health”), were *facilitators* (e.g. “the warm weather supports the claimant’s recovery”), *no problem* (e.g. “the weather does not influence the claimant’s health”), or *facts* (e.g. “the weather is usually mild where the claimant lives”).

Concepts not appropriately classifiable with the ICF were labeled with a specification code as either *personal factors*, *not covered*, *not definable (broad concepts)* or *health condition*. The codes *other specified*, *not definable (within ICF components)* or *combination category* were applied when concepts could not be addressed sufficiently precisely within ICF categories. Table 2 provides examples and definitions for the different types of *specification categories*.

**Table 2:** **Specification areas, type of specification categories, examples for specification codes and definitions for the different types of specification categories referring to concepts in the medical reports not appropriately codeable with the ICF**

Specification area	Type of specification categories and examples for specification codes	Definition of specification category
*(1) Precision*	Combination category	Location of a body function
	e.g. b7101(s7201)	e.g. mobility of shoulder joint
	Not definable (within ICF components)	Concepts which can be coded to more than one ICF category within a component
	e.g. nd-d(ohw)	e.g. overhead working
	Other specified	Concepts not differentiable within an ICF category
	e.g. d4158	e.g. maintaining a bending position
*(2) Coverage*	Not covered	Not covered by the ICF
	e.g. nc-acc	e.g. accidents
	Not covered – work (in)capacity	Not covered within the ICF (general work (in)capacity)
	e.g. nc-WC; nc-WIC	e.g. work (in)capacity
	Health condition	Health conditions
	e.g. hc	e.g. depression
*(3) Personal factors*	Personal factors	Personal factors
	e.g. pf-edu	e.g. educational background
*(4) Broad concepts*	Not definable (broad concepts)	Not definable broad concepts
	e.g. nd-gh	e.g. general health

We assigned the categories to the four specification areas *precision*, *coverage*, *personal factors* and *broad concepts* (see Table 2). Information referring to causal relationships, consistency or time-related aspects was not considered for the content analysis.

### Relevance analysis

The relevance analysis only included *specification categories* and *third or fourth level ICF categories* assessed as *limitations*, *barriers* or *facilitators* and thus assumed to influence the claimant’s functioning. We removed second level ICF categories identified in our previous study [27] from the analysis. In addition, we excluded concepts from the specification area *coverage* referring to *health condition* or to the legal term *work (in)capacity* and *broad concepts*. The former because a health condition such as depression can be classified using an ICD-10 code. The latter two since classifying overall concepts would not match the ICF’s basic tenet and is not further increasing a comprehensive and accurate reporting of functioning aspects and their determinants in MEWC.

**Figure 2:**
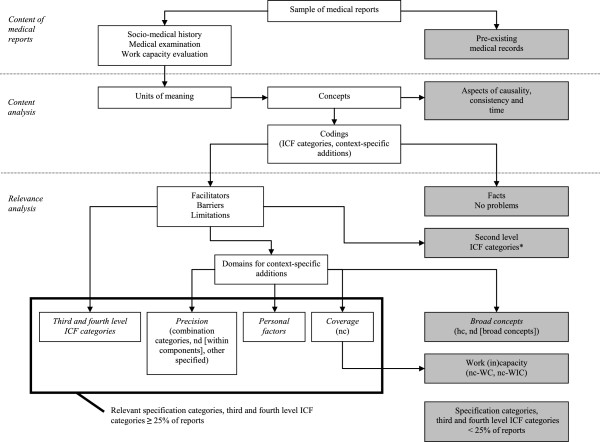
**Overview of the content selection process.** Notes: * = Second level ICF categories already identified in a previous study [27]; hc = health condition; nd = not definable with the ICF; nc = not covered by the ICF; WC = work capacity; WIC = work incapacity. Boxes shaded in grey refer to concepts that were excluded in the content selection process.

We operationalized the *relevance* of a category as its *relative frequency* across reports, i.e. the percentage of reports it was addressed in at least once, and applied a *relevance threshold* at 25%. All categories above this threshold were considered relevant context-specific additions to the ICF for MEWC involving CWP and LBP. The selection of a particular threshold always involves an arbitrary element. For the purpose of this study, we first analyzed the frequency and diversity of the categories with regard to three different thresholds, i.e. 75%, 50% and 25%. We eventually settled on the most lenient threshold at 25% so as to arrive at a comprehensive picture of the relevant context-specific additions across reports.Figure 2 illustrates the content selection process applied in our study.

## Results

### Sample characteristics

The saturation criterion was reached after coding 30 medical reports. However, to fulfill the heterogeneity requirement, we set the minimum size of the subsample to be 72 reports, representing about one third of the available sample of 209 reports. We considered this sample size big enough to ensure a proportional inclusion of the index conditions and the medical disciplines involved in the reports. 27 of the reports contained only a CWP diagnosis, 22 only a LBP diagnosis, and 23 both a CWP and LBP diagnosis. In the CWP group 20 reports included one, 4 two and 26 more than two medical disciplines, while in the LBP group 14 reports consisted of one, 5 of two and 26 of more than two disciplines. In both group of reports, psychiatry and rheumatology were the most frequent medical disciplines.

### Interrater reliability

The percentage agreement between the two coders was 78.7% for the ICF categories and 78.8% for the specification categories in the reports.

### Reports with CWP diagnoses

#### Content analysis

A total of 21,562 units of meaning led to 45,365 (100%) codings. Out of these, 24,396 (53.7%) represented pure ICF categories. The rest (20,969 or 46.3%) was not classifiable appropriately or precisely enough with the ICF. Table 3 displays the codings’ frequencies for the different types of specification categories and specification areas.

**Table 3:** **Absolute and relative frequency of the codings for the different types of specification categories and specification areas in relation to the total number of codings (k = 45,365) in the CWP reports (n = 50)**

Specification area	Type of specification categories	Absolute frequency	Relative frequency %
*(1) Precision*	Combination categories	4,775	10.5
	Not definable (within components)	592	1.3
	Other specified	871	1.9
		*6,238*	*13.8*
*(2) Coverage*	Not covered	3,324	7.3
	Not covered - work (in)capacity	770	1.7
	Health condition	2,243	4.9
		*6,337*	*13.9*
*(3) Personal factors*	Personal factors	*4,276*	*9.4*
*(4) Broad concepts*	Not definable (broad concepts)	*4,118*	*9.1*

Of the 24,396 (100%) codings referring to ICF categories, 8,413 (34.5%) were coded on the second, 13,810 (56.6%) on the third and 2,173 (8.9%) on the fourth level.

#### Relevant specification categories

Overall, 5,146 codings for specification categories and 5,482 for third and fourth level ICF categories were assessed as limitations, barriers or facilitators and thus included in the relevance analysis. 454 *different* specification categories were identified. Forty-three of them passed the 25%-threshold and were considered relevant for medical reports on claimants with CWP. Thirty-one categories belonged to the area precision, 10 to personal factors and 2 to coverage (see Table 4). In addition, 70 third or fourth level ICF categories passed the relevance threshold (see Table 5).

**Table 4:** **Relative frequency of the specification categories (subdivided in specification areas) in the CWP reports (n = 50)**

Specification area	Code	Specification category	Relative frequency %
*(1) Precision*	nd-d(hph-l)	Heavy physical labor	60
	b28016(s76002)	Pain in joints (Lumbar vertebral column)	56
	b7101(s76002)	Mobility of several joints (Lumbar vertebral column)	56
	b2801(s7601)	Pain in body part (Muscles of trunk)	54
	b2801(s740)	Pain in body part (Structure of pelvic region)	48
	b2801(s720)	Pain in body part (Structure of shoulder region)	46
	b28016(s76000)	Pain in joints (Cervical vertebral column)	46
	b7101(s76000)	Mobility of several joints (Cervical vertebral column)	46
	b2801(s710)	Pain in body part (Structure of head and neck region)	44
	b28015(s7500)	Pain in lower limb (Structure of thigh)	44
	b1268	Temperament and personality functions, other specified (Aggravation, simulation)	44
	b2702(s730)	Sensitivity to pressure (Structure of upper extremity)	42
	b28016(s75011)	Pain in joints (Knee joint)	42
	b2803(s750)	Radiating pain in a dermatome (Structure of lower extremity)	42
	b7350(s75002)	Tone of isolated muscles and muscle groups (Muscles of thigh)	42
	b2702(s750)	Sensitivity to pressure (Structure of lower extremity)	38
	b28016(s7201)	Pain in joints (Joints of shoulder region)	38
	d2408	Handling stress and other psychological demands, other specified (Behavior during medical examination)	38
	s76082	Structure of trunk, other specified (Lumbar intervertebral disk)	38
	b7350(s7601)	Tone of isolated muscles and muscle groups (Muscles of trunk)	36
	nd-d(ohw)	Overhead working	34
	nd-d(fp)	Forced postures	34
	b28015(s7501)	Pain in lower limb (Structure of lower leg)	32
	b28014(s7302)	Pain in upper limb (Structure of hand)	30
	b28016(s76001)	Pain in joints (Thoracic vertebral column)	30
	b2803(s7500)	Radiating pain in a dermatome (Structure of thigh)	30
	b7101(s75001)	Mobility of several joints (Hip joint)	28
	b7101(s7401)	Mobility of several joints (Joints of pelvic region)	26
	b7101(s76001)	Mobility of several joints (Thoracic vertebral column)	26
	nd-d(rep)	Repetitive work activities	26
	e1108	Products or substances for personal consumption, other specified (Stimulants like alcohol or nicotine)	26
*(2) Coverage*	nc-fam	Genetic aspects	70
	nc-acc	Accidents	58
*(3) Personal factors*	pf-othchar	Other personal characteristics (e.g. personal expectations, beliefs and attitudes)	90
	pf-exp	Past and current experience (past life events and concurrent events)	88
	pf-edu	Education	84
	pf-fam	Family and marital status	84
	pf-psychassets	Individual psychological assets	76
	pf-copstyles	Coping styles	70
	pf-lifestyle	Lifestyle	62
	pf-socbac	Social background	58
	pf-char	Overall behavior pattern and character style	52
	pf-prof	Profession	52

**Table 5:** **Relative frequency of the third and fourth level ICF categories in the CWP reports (n = 50)**

ICF code	Third or fourth level ICF category	Relative frequency %
e5800	Health services	88
b28013	Pain in back	86
b28016	Pain in joints	84
e1101	Drugs	82
b1265	Optimism	78
b28015	Pain in lower limb	76
b28010	Pain in head and neck	76
b1602	Content of thought	74
b7101	Mobility of several joints	74
b2702	Sensitivity to pressure	70
b2803	Radiating pain in a dermatome	70
s7600	Structure of vertebral column	70
d5702	Maintaining one's health	70
b1342	Maintenance of sleep	68
e1650	Financial assets	68
d8700	Personal economic resources	64
b1301	Motivation	60
b1603	Control of thought	60
d4153	Maintaining a sitting position	60
b28014	Pain in upper limb	58
b7350	Tone of isolated muscles and muscle groups	58
s76002	Lumbar vertebral column	58
b2802	Pain in multiple body parts	56
b4552	Fatiguability	56
b1521	Regulation of emotion	54
b1303	Craving	52
s7502	Structure of ankle and foot	48
b1261	Agreeableness	48
d4300	Lifting	48
e5702	Social security policies	48
b1302	Appetite	46
d4150	Maintaining a lying position	46
d7701	Spousal relationships	46
s76001	Thoracic vertebral column	46
d8450	Seeking employment	44
d2402	Handling crisis	44
e5700	Social security services	44
d2303	Managing one's own activity level	42
b2703	Sensitivity to noxious stimulus	40
b2800	Generalized pain	40
b1470	Psychomotor functions	40
d4154	Maintaining a standing position	40
d8502	Full-time employment	38
b1520	Appropriateness of emotion	38
d7601	Child–parent relationships	38
d7602	Sibling relationships	38
b7301	Power of muscles of one limb	36
d8501	Part-time employment	36
b28012	Pain in stomach or abdomen	36
b4200	Increased blood pressure	36
d4501	Walking long distances	36
b7300	Power of isolated muscles and muscle groups	34
b1522	Range of emotion	34
b7305	Power of muscles of the trunk	34
d2401	Handling stress	34
d7600	Parent–child relationships	34
s76003	Sacral vertebral column	34
s7501	Structure of lower leg	32
e2450	Day/night cycles	32
s76000	Cervical vertebral column	32
b1263	Psychic stability	30
b1341	Onset of sleep	30
b7355	Tone of muscles of trunk	30
d4104	Standing	30
d4751	Driving	30
e2250	Temperature	30
b1260	Extraversion	26
b1266	Confidence	26
d5701	Managing diet and fitness	26
d7202	Regulating behaviors within interactions	26

### Reports with LBP diagnoses

#### Content analysis

A total of 21,707 units of meaning resulted in 42,116 (100%) codings. Out of these, 22,333 (53%) represented pure ICF categories. The remainder (19,783 or 47%) was not classifiable appropriately or precisely enough with the ICF. Table 6 presents the codings’ frequencies for the different types of specification categories and specification areas.

**Table 6:** **Absolute and relative frequency of the codings for the different types of specification categories and specification areas in relation to the total number of codings (k = 42,116) in the LBP reports (n = 45)**

Specification area	Type of specification categories	Absolute frequency	Relative frequency %
*(1) Precision*	Combination categories	5,297	12,6
	Not definable (within components)	623	1.5
	Other specified	1,246	3.0
		*7,166*	*17.0*
*(2) Coverage*	Not covered	2,568	6.1
	Not covered – work (in) capacity	754	1.8
	Health condition	2,571	6.1
		*5,893*	*14.0*
*(3) Personal factors*	Personal factors	*3,111*	*7.4*
*(4) Broad concepts*	Not definable (broad concepts)	*3,613*	*8.6*

Of the 22,333 (100%) codings referring to ICF categories, 6,712 (30.1%) were coded on the second, 12,588 (56.4%) on the third and 3,033 (13.6%) on the fourth level.

#### Relevant specification categories

Overall, 4,860 codings for specification categories and 5,184 for third and fourth level ICF categories were assessed as limitations, barriers or facilitators and thus included in the relevance analysis. 438 *different* specification categories were identified. Fifty-two of them passed the 25%-threshold and were considered relevant for medical reports on claimants with LBP. Forty categories belonged to the area precision, 10 to personal factors and 2 to coverage (see Table 7). In addition, 67 third or fourth level ICF categories passed the relevance threshold (see Table 8).

**Table 7:** **Relative frequency of the specification categories (subdivided in specification areas) in the LBP reports (n = 45)**

Specification area	Code	Specification category	Relative frequency %
*(1) Precision*	b7101(s76002)	Mobility of several joints (Lumbar vertebral column)	84
	b28016(s76002)	Pain in joints (Lumbar vertebral column)	80
	s76082	Structure of trunk, other specified (lumbar intervertebral disks)	76
	b7101(s76002)	Mobility of several joints (Lumbar vertebral column)	69
	b2803(s750)	Radiating pain in a dermatome (Structure of lower extremity)	64
	nd-d(hph-l)	Heavy physical labor	64
	b7350(s75002)	Tone of isolated muscles and muscle groups (Muscles of thigh)	58
	b2801(s7601)	Pain in body part (Muscles of trunk)	56
	b28016(s7201)	Pain in joints (Joints of shoulder region)	51
	b7350(s7601)	Tone of isolated muscles and muscle groups (Muscles of trunk)	49
	b2803(s7500)	Radiating pain in a dermatome (Structure of thigh)	47
	s76083	Structure of trunk, other specified (sacral intervertebral disks)	47
	b7101(s7401)	Mobility of several joints (Joints of pelvic region)	42
	nd-d(fp)	Forced postures	42
	b7101(s76001)	Mobility of several joints (Thoracic vertebral column)	40
	nd-d(ohw)	Overhead working	40
	b7101(s75001)	Mobility of several joints (Hip joint)	36
	b7101(s7600)	Mobility of several joints (Structure of vertebral column)	36
	b1268	Temperament and personality functions, other specified (Aggravation, simulation)	36
	d2408	Handling stress and other psychological demands, other specified (Behavior during examination)	36
	d4158	Maintaining a body position, other specified (Maintaining a bending position)	36
	b28016(s76001)	Pain in joints (Thoracic vertebral column)	33
	b2803(s710)	Radiating pain in a dermatome (Structure of head and neck region)	33
	b2803(s7502)	Radiating pain in a dermatome (Structure of ankle and foot)	33
	b7108	Mobility of joint functions, other specified (Single multiple joint)	33
	d4108	Changing a basic body position, other specified (Back or head rotations)	33
	b28016(s75011)	Pain in joints (Knee joint)	31
	s76080	Structure of trunk, other specified (Cervical intervertebral disks)	31
	b2702(s7302)	Sensitivity to pressure (Structure of hand)	29
	b2702(s750)	Sensitivity to pressure (Structure of lower extremity)	29
	b2801(s7104)	Pain in body part (Muscles of head and neck region)	29
	b2801(s720)	Pain in body part (Structure of shoulder region)	29
	b750(s75012)	Motor reflex functions (Muscles of lower leg)	29
	b2702(s730)	Sensitivity to pressure (Structure of upper extremity)	27
	b28014(s7302)	Pain in upper limb (Structure of hand)	27
	b28016(s75001)	Pain in joints (Hip joint)	27
	b2803(s730)	Radiating pain in a dermatome (Structure of upper extremity)	27
	b7301(s750)	Power of muscles of one limb (Structure of lower extremity)	27
	nd-d(rep)	Repetitive work activities	27
	nd-d(altact)	Alternating work activities	27
*(2) Coverage*	nc-fam	Genetic aspects	56
	nc-acc	Accidents	51
*(3) Personal factors*	pf-fam	Family and marital status	73
	pf-exp	Past and current experience (Past life events and concurrent events)	69
	pf-psychassets	Individual psychological assets	67
	pf-othchar	Other personal characteristics (e.g. personal expectations, beliefs and attitudes)	62
	pf-copstyles	Coping styles	56
	pf-edu	Education	56
	pf-prof	Profession	47
	pf-lifestyle	Lifestyle	44
	pf-char	Overall behavior pattern and character style	36
	pf-socbac	Social background	36

**Table 8:** **Relative frequency of the third and fourth level ICF categories in the LBP reports (n = 45)**

ICF code	Third or fourth level ICF category	Relative frequency %
b28013	Pain in back	100
b28016	Pain in joints	98
b7101	Mobility of several joints	98
s7600	Structure of vertebral column	98
s76002	Lumbar vertebral column	93
b2803	Radiating pain in a dermatome	89
d4153	Maintaining a sitting position	89
b7350	Tone of isolated muscles and muscle groups	82
b28010	Pain in head and neck	78
s76001	Thoracic vertebral column	78
e1101	Drugs	76
b28015	Pain in lower limb	76
e5800	Health services	76
b2702	Sensitivity to pressure	71
d4300	Lifting	71
d4154	Maintaining a standing position	64
e1650	Financial assets	62
d8700	Personal economic resources	58
b1342	Maintenance of sleep	58
d4150	Maintaining a lying position	58
b1265	Optimism	56
b4200	Increased blood pressure	56
s76000	Cervical vertebral column	53
s76003	Sacral vertebral column	53
d5702	Maintaining one's health	53
e5702	Social security policies	53
b28014	Pain in upper limb	51
b1602	Content of thought	49
b1303	Craving	47
s7502	Structure of ankle and foot	47
b7301	Power of muscles of one limb	47
d8450	Seeking employment	47
b1301	Motivation	44
d8501	Part-time employment	44
d4105	Bending	44
s7501	Structure of lower leg	42
b4552	Fatiguability	42
b2802	Pain in multiple body parts	42
b7305	Power of muscles of the trunk	42
b1603	Control of thought	40
b1521	Regulation of emotion	40
d4104	Standing	40
d4501	Walking long distances	40
b1261	Agreeableness	38
d8502	Full-time employment	38
e5700	Social security services	38
b1302	Appetite	38
e2450	Day/night cycles	36
d4551	Climbing	36
b1470	Psychomotor control	33
b7300	Power of isolated muscles and muscle groups	33
b28012	Pain in stomach or abdomen	33
b7355	Tone of muscles of trunk	33
s1201	Spinal nerves	31
d2303	Managing one's own activity level	31
b2703	Sensitivity to a noxious stimulus	31
d7701	Spousal relationships	31
b1341	Onset of sleep	31
d7602	Sibling relationships	31
d7601	Child–parent relationships	31
s75011	Knee joint	29
e2250	Temperature	29
b7303	Power of muscles in lower half of the body	29
b7100	Mobility of a single joint	27
s75021	Ankle joint and joints of foot and toes	27
d2401	Handling stress	27
d5701	Managing diet and fitness	27
d2402	Handling crisis	27

## Discussion

We identified several potential context-specific additions to the ICF taxonomy for its application in MEWC involving CWP and LBP. Moreover, we found a substantial number of third and fourth level ICF categories to be relevant for this particular context. The specification categories were assigned to the four specification areas *precision*, *coverage*, *personal factors* and *broad concepts*. For reasons given in the methods section, the categories referring to broad concepts were not considered relevant context-specific additions for the use of the ICF in MEWC.

*Precision* was in both groups of reports the most common specification area and reflects category specifications that need to be considered when applying the ICF for comprehensive reporting in MEWC involving CWP and LBP. For instance, the ICF category for joint pain (i.e. *b28016 Pain in joints*) lacks granularity in MEWC as it does not distinguish between different locations of joint pain. Such a differentiation, however, is important in MEWC as joint pain may affect different work activities depending on its location. While pain in the lumbar vertebral column, for example, may limit bending, shoulder pain interferes with activities requiring hand and arm use. Category specifications for different locations of joint pain could facilitate an accurate reporting of such relations when using the ICF in MEWC. In addition, important work activities such as overhead working involve aspects covered by several different ICF categories and cannot be adequately reported with one single ICF category. This problem could be resolved by introducing context-specific additions to the ICF addressing the work activities concerned.

The large number of third or fourth level ICF categories above the relevance threshold indicates their importance when applying the ICF in MEWC involving chronic pain. This is particularly true for describing pain locations on the one hand and work activities on the other, and is consistent with findings of studies in the contexts of work and pain assessments [15, 16].

As to a potential lack of specificity of ICF categories, it needs to be emphasized that the ICF taxonomy aims at providing a universally applicable standard for reporting health and functioning information rather than at offering accurate categories for reporting specific aspects of particular disciplines, contexts or health conditions. In this respect, our findings regarding a lack of specificity of some ICF categories are not surprising, but with regard to the potential applicability of the ICF in the present context nevertheless noteworthy. Alternative to the use of context-specific additions it is also possible to report aspects that cannot be accurately classified with ICF categories by using free text and without applying specific ICF-related codes.

*Personal factors* are the second important area for context-specific additions to the ICF in MEWC involving CWP and LBP. Standardized reporting of psychological aspects such as coping strategies or pain beliefs [23] as well as occupational experiences or work motivation [24] is crucial for pain and work ability assessments. In MEWC, personal factor categories may be helpful in illustrating whether functional limitations are likely due to a health condition (e.g. “depressive symptoms”) or due to individual characteristics (e.g. “reduced work motivation”). Whilst in the former case claimants may be entitled to receive a disability pension, in the latter they are more likely to be assigned to a return to work program. Most relevant personal factors in the context of MEWC involving chronic pain were found to be the claimant’s educational, occupational and biographical background, behavior patterns as well as personal emotions and cognitions such as, for instance, expectations related to the job [25]. As an alternative to the determination of context-specific additions for personal factors, already existing personal factor taxonomies could be used for standardized reporting such as the one of *Geyh et al*. [37] or *Grotkamp et al*. [38]. These taxonomies have recently been applied for coding the content of MEWC involving CWP [25].

In both groups of reports only two categories from the area *coverage* passed the relevance threshold (i.e. *genetic aspects* and *accidents*). This is a good sign regarding the comprehensiveness and exhaustiveness of the ICF in covering the content of MEWC involving chronic pain, and an indicator that adding context-specific aspects not covered by the ICF is a less pressing issue.

### Study limitations

Our study has some limitations. First, our sample only includes reports in German from the Swiss national disability insurance. Results are thus not generalizable to other insurance schemes nor to countries with different disability evaluation procedures. To test the generalizability of our findings in other insurance schemes or in other national contexts, further validation studies would be required.

Second, in our study we considered medical reports as the gold standard and benchmark for the comprehensiveness of the ICF in capturing the core content of MEWC. However, it is possible that these reports do not address all aspects that are relevant for MEWC in a sufficiently comprehensive manner. Moreover, it is unknown to what extent the information in these reports addresses the subjective experience of the claimants in an unfiltered, uninterpreted and truly person-centered manner. With the application of a rather lenient relevance threshold at 25%, we increased the probability of capturing a comprehensive picture of the relevant aspects across reports and, thus, alleviated the former of these two limitations. However, additional data sources such as interviews with experts or claimants should be considered to validate our findings.

### Practical implications

The context-specific additions to the ICF and the third or fourth level ICF categories we established as relevant for MEWC involving CWP and LBP complete the set of second level categories suggested in our previous study [27]. Our findings are exclusively geared toward the application of the ICF in MEWC involving CWP or LBP and do not represent suggestions for a general adjustment of the ICF taxonomy or for its use in other contexts. Comprehensive documentation based on the ICF categories and the context-specific additions identified in our studies could ensure transparent MEWC and standardize them in terms of what to measure. However, the ICF does not currently provide a proper operationalization for its categories. Therefore, the issue of how to measure the identified categories should be addressed for the time being by assigning validated measurement tools to the categories.

It is obvious that comprehensive reporting involves a considerable amount of categories, which threatens to undermine the practicability of MEWC. In Switzerland, MEWC of claimants with CWP are usually conducted in multidisciplinary settings. To ensure feasible evaluations, the categories could be grouped and assigned to the particular discipline in charge of their assessment (e.g. *b152 Emotional functions* should be exclusively assessed by psychiatrists). This limits the amount of categories to be assessed by each medical expert and ensures an overall documentation structure of the multidisciplinary evaluations.

## Conclusions

The ICF taxonomy represents a universally applicable standard for reporting health and functioning information. However, when applying the ICF for comprehensive and transparent reporting in MEWC involving CWP and LBP context-specific additions are needed. This is particularly true for the documentation of specific pain-related issues, work activities and personal factors. To ensure the practicability of the multidisciplinary evaluation process, the large number of ICF categories and context-specific additions necessary for comprehensive documentation could be specifically allocated to the disciplines in charge of their assessment.

## Abbreviations

MEWC: Medical evaluations of work capacity
ICF: International Classification of Functioning, Disability and Health
CWP: Chronic widespread pain
LBP: Low back pain
ICD: International Classification of Diseases.
